# Evaluating the Color Matching Ability of a Smart Chromatic Technology–Based Composite Resin for Premolar Teeth Restoration

**DOI:** 10.1155/2024/5514821

**Published:** 2024-09-27

**Authors:** Kamyar Fathpour, Alaleh Salehi, Pouran Samimi, Amirhossein Fathi

**Affiliations:** ^1^ Department of Restorative Dentistry Dental Materials Research Center Dental Research Institute School of Dentistry Isfahan University of Medical Sciences, Isfahan, Iran; ^2^ Dental Students Research Committee School of Dentistry Isfahan University of Medical Sciences, Isfahan, Iran; ^3^ Operative Dentistry Department Dental Materials Research Centre Dental Research Institute School of Dentistry Isfahan University of Medical Sciences, Isfahan, Iran; ^4^ Dental Prosthodontics Department Dental Materials Research Center School of Dentistry Isfahan University of Medical Sciences, Isfahan, Iran

**Keywords:** color matching, color measurement, composite resin

## Abstract

**Objectives:** This study aims to evaluate the color-matching ability of OMNICHROMA composite, a single-shade composite, using the CIE L^*∗*^a^*∗*^b^*∗*^ system and determining *Δ*E values.

**Materials and Methods:** In this in vitro study, 30 intact premolar teeth were collected, cleaned, and disinfected with 0.5% thymol solution. The teeth were stored in distilled water at 37°C. A putty index calibrated the color assessment sites on buccal and lingual surfaces. Two cavities were prepared, one with 1.25 mm depth (enamel) and another with 2.25 mm depth (dentin), and restored using OMNICHROMA composite resin. The putty index measured the color of the cured composite, and *Δ*E between the composite and original tooth color was calculated.

**Statistical analysis:** Data analysis included *t*-tests, correlation coefficients, and Fisher's exact test (*α* = 0.05).

**Results:** The mean *Δ*E was 2.39 in enamel cavities and 2.32 in dentin cavities, both within the clinically acceptable range (<3.3). In enamel cavities, the composite color was darker than the tooth, shifting toward green and blue shades. In dentin cavities, the composite color was lighter than the tooth, with shifts toward green and yellow shades. Color matching was slightly better in dentin cavities, but the difference was not statistically significant (*p*=0.719).

**Conclusion:** OMNICHROMA composite resin's color matching falls within the clinically acceptable range, unaffected by cavity depth.

## 1. Introduction

The increasing demand for cosmetic dentistry has driven rapid advancements in restorative resin composites [1, 2]. For over 40 years, dentists have used these composites as direct restorative materials, making them a viable alternative to amalgam and gold restorations [3]. These materials come in various forms and colors, with selecting the right color being a critical step in their application [4]. Just as the shape of teeth is crucial for pleasing facial esthetics, so is the color, which significantly affects the appearance of the final restoration. Sometimes, the specific color of a tooth and the structure of dental pigments can lead to color mismatches [5]. Additionally, there are slight color variations among different parts of a tooth. Consequently, tooth color and appearance are complex phenomena influenced by factors such as environmental lighting, translucency, opacity, and light scattering within and through the tooth, as well as visual perception and brain processing, collectively forming the overall perception of the tooth color [6].

When choosing the color of composite restorations, factors that might lead to color changes over time must be carefully considered to ensure durability and color stability. This adds complexity to the dentist's responsibilities, as even minor errors can yield unsatisfactory outcomes [7, 8]. Achieving color harmony between the composite material and the natural dental structure is one of the foremost concerns for patients [9]. In recent years, substantial efforts have been made to achieve optimal esthetic results with restorative materials, aiming to replicate the characteristics of natural teeth, including color, translucency, surface texture, and long-term color stability [10]. Consequently, dental materials with the ability to adapt their color to match the surrounding hard tissues offer significant clinical advantages. They can enhance esthetic qualities, streamline color-matching procedures, and to some extent, mitigate color disparities, thus reducing the reliance on extensive shade guides [11, 12].

Various color systems have been introduced for selecting the color of tooth-colored restorations. The CIE color display system, derived from the French name Commission Internationale de l'Éclairage (International Commission on Illumination), is designed for color measurement purposes. This globally accepted system precisely defines the characteristics of any color and is widely utilized by reputable scientific institutions. The CIE L^*∗*^a^*∗*^b^*∗*^ system (the color space system) is a valuable method for consistently obtaining information about the position of a color in a three-dimensional color space. This system defines color using three axes: L^*∗*^, a^*∗*^, and b^*∗*^. The L^*∗*^ index represents luminance or brightness, whilea^*∗*^ and b^*∗*^ serve as numerical axes for hue and chroma [13].

Previous studies assessing the color-matching of composite resins primarily used acrylic teeth or designed Class 1 cavities [11, 14]. Additionally, some studies relied solely on visual observation to assess color matching [15]. Furthermore, the influence of depth as a factor in color matching was not investigated in any of these studies. OMNICHROMA composite stands out as a universal single-shade composite material that claims to mimic tooth color effectively in many clinical cases [9]. Therefore, it is essential to assess this composite's color-matching capability. This study aims to evaluate the color-matching ability of the OMNICHROMA composite using the CIE L^*∗*^a^*∗*^b^*∗*^ system through the measurement of *Δ*E values.

## 2. Sample Collection and Preparation

In this in vitro study design, 30 premolar teeth without color changes, cavities, or fractures were collected, having been extracted for orthodontic reasons or periodontal problems (*α* = 0.05). Blood and tissue remnants were removed using a scalpel blade. The teeth were stored in a 5% thymol solution for 1 week and subsequently kept in distilled water at 37°C until the preparation procedure. Ethical approval was obtained from the Ethics Committee of Isfahan University of Medical Sciences and Health Services (ethical code: IR.MUI.RESEARCH.REC.1401.153). The teeth were collected from patients who voluntarily donated their extracted teeth for research and educational purposes.

## 3. Evaluation of Tooth Color Before Preparation

Prior to color measurement, all teeth were cleaned using a rubber cup prophylaxis with a low-speed handpiece, employing a combination of fluoride-free pumice and water, followed by rinsing with water. The initial color of the premolars was recorded using the Vita shade guide, ensuring a range of shades for a comprehensive analysis of the color blending efficacy of OMNICHROMA. The lab values collected for the 30 teeth corresponded to shades within the A1–A3 range of the Vita shade guide, primarily representing light shades. The Shade Pilot device (DeguDent, Hanau, Germany) was calibrated using built-in white and green plates.

**Figure 1:**
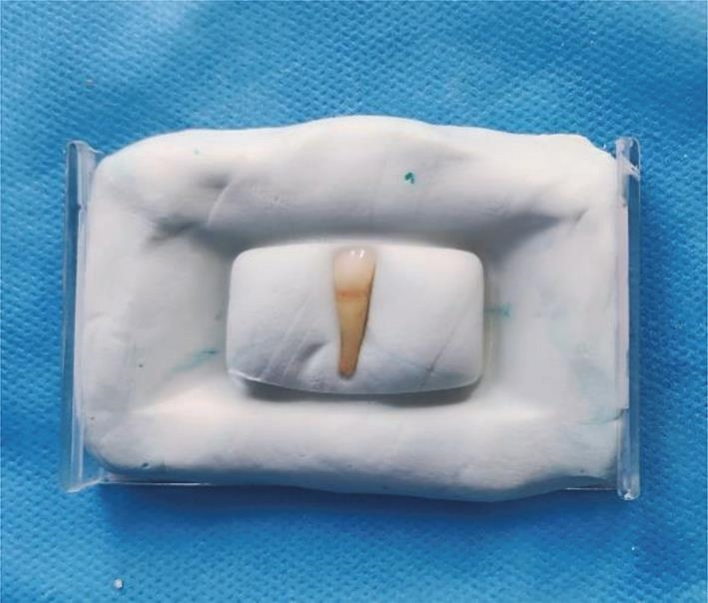
Tooth preparation (tooth mounted in putty).

To ensure consistent color measurements, the same anatomical landmarks on each tooth were used. A customized mold was created for each tooth to ensure correct positioning during consecutive color measurements. Initially, the inner surface of a clear plastic box was coated with Vaseline. Subsequently, putty and activator of a condensation silicone (Speedex, Coltene, Switzerland) were mixed and placed inside the mold. The Shade Pilot device was fixed to detect the tooth during successive color measurements (Figure 1). This ensured consistent device placement at a specific location for each color determination, minimizing errors due to varying tooth positions. This step was repeated for each tooth on both the buccal and lingual surfaces. The putty index positioned either the buccal or lingual surfaces of the teeth in the center of the index, ensuring reported numbers were not affected by sample misalignment. All teeth underwent color determination using the Shade Pilot, with all measurements repeated three times.

## 4. Tooth Preparation

The buccal surface was selected for preparing shallow enamel cavities, while the lingual surface was chosen for deep dentin cavities. Cavity depths were standardized using a calibrated dental handpiece with depth-specific burs and a digital caliper for measurement. An electronic digital vernier caliper (aerospace 0–150 mm with a resolution of 0.01 mm) was used to measure and verify cavity depths during and after preparation to ensure consistency.

Standard cavities were prepared in two groups using an occlusal reduction bur (Meisinger, Centennial, CO, United States). In the buccal cavities, a cavity with a depth of 1.25 mm and 1.5 mm width was created using an 828 w bur. In the lingual cavity, a cavity with a depth of 2.25 mm and 1.5 mm width was created. Each cavity was made with a single entry of the bur into the relevant area, with each bur used to prepare six cavities. Dentin cavities were etched with phosphoric acid (Pulpdent, Watertown, MA, United States) for 20 s, then rinsed thoroughly with an air–water spray for 20 s, and air-dried until a frosted appearance was observed on the enamel margin of the cavity. The bonding system Palfique bond (Tokuyama, Tokyo, Japan) was applied according to the manufacturer's instructions with a microbrush for 10 s. Gentle air pressure was applied until the bonding layer was immobilized, and it was light cured with a light intensity of 800 mW/cm^2^ (Mectron, Carasco, Italy).

Cavities were restored with OMNICHROMA resin composite (Tokuyama, Tokyo, Japan) incrementally, with each layer light cured for 20 s. Restorations were polished using a diamond flame-shaped bur (Teeskavan, Hashtgerd, Iran) and completed with Enhance polishing disks (Enhance, Milan, Italy). Each restoration was polished for 15 s, and postcuring was performed for 5 s with a light intensity of 1200 mW/cm^2^. All processes were performed separately for each sample by one operator. Samples were stored in distilled water for 24 h before color measurement.

## 5. Composite Color Evaluation

The cured composite color was assessed using the Shade Pilot color measurement device, with all measurements repeated three times. Each sample's image before and after preparation remained in the device's memory with a unique code. Before color measurement of the composite restoration, the corresponding sample code was selected in the device, and the area of interest was identified using the device's pen, displaying *Δ*E and all L, a, and b factors before and after the procedure.

## 6. Statistical Analysis

The data obtained from the Shade Pilot device were analyzed using SPSS 27 (IBM, Armonk, NY, United States) software. Initially, the mean and standard deviation were calculated for each parameter. A paired *t*-test was performed to compare each parameter before and after restoration according to cavity depth. An independent *t*-test was also carried out to assess differences in optical parameters between different groups (*p* value < 0.05). To examine the linear relationship between different parameters, a table of correlation coefficients was used, and correlation coefficients (*r* values) and *p* values were calculated.

## 7. Results

The measurements for the L^*∗*^, a^*∗*^, and b^*∗*^ parameters before and after restoration are summarized in Table 1. For the enamel group, the average L^*∗*^ value decreased significantly after restoration, indicating that the composite color exhibited less brightness compared to the tooth (*p*  < 0.001). The average a^*∗*^ value also decreased significantly, suggesting a shift toward a greener hue (*p*  < 0.001). The b^*∗*^ value average decreased, implying a shift toward blue, though this change was not statistically significant (*p*=0.775).

In the dentin group, the average L^*∗*^ value increased after restoration, indicating a slight increase in brightness, but this change was not statistically significant (*p*=0.989). The average a^*∗*^ value decreased significantly, showing a shift toward green (*p*  < 0.001). The b^*∗*^ value average increased, suggesting a shift toward yellow, though this was not statistically significant (*p*=0.137).

The *∆*E values in the dentin group were lower than in the enamel group, but this difference was not statistically significant (*p*=0.719). Both groups' *∆*E values were within the clinically acceptable range (*∆*E < 3.3) (Table 2).

For detailed optical parameter analysis, Table 3 presents the comparison of L^*∗*^, a^*∗*^, and b^*∗*^ values between the enamel and dentin groups. No statistically significant differences were observed in L^*∗*^ (*p*=0.7), a^*∗*^ (*p*=0.29), and b^*∗*^ (*p*=0.12) parameters between the two groups.

Correlation analysis (Table 4) revealed a strong linear relationship between *∆*E and *∆*L in the enamel group. In contrast, in the dentin group, *∆*E showed a moderate linear relationship with *∆*L and a strong linear relationship with *∆*b. This suggests that color mismatch in enamel is primarily influenced by changes in brightness (*∆*L), whereas in dentin, the mismatch is more related to changes in the blue–yellow axis (*∆*b).

Table 5 shows no significant linear relationship between initial L^*∗*^, a^*∗*^, and b^*∗*^ values and *∆*E. This indicates that initial tooth color parameters do not predict the degree of color match for the composite.

Table 6 displays the frequency distribution of *∆*E values in relation to the clinically acceptable threshold of 3.3. The Fisher exact test showed no significant difference between the enamel and dentin groups (*p*=0.472), with 85% of samples falling within the clinically acceptable range.

## 8. Discussion

This study aimed to evaluate the color match of OMNICHROMA composite resin in restored teeth with varying cavity depths. The results indicate that although statistically significant differences were found in some color parameters, the overall *∆*E values for both enamel and dentin restorations were within the clinically acceptable range (*∆*E < 3.3).

In the enamel group, the significant decrease in L^*∗*^ (brightness) and a^*∗*^ (greenness) suggests that the composite restoration was less bright and more green compared to the natural tooth. However, the lack of significant change in b^*∗*^ (blue–yellow axis) indicates that the shift toward blue was not statistically supported.

In the dentin group, while there was a significant decrease in a^*∗*^ (greenness) and an increase in b^*∗*^ (yellow), the changes in brightness (L^*∗*^) were not statistically significant. This suggests that the color match for dentin restorations is less affected by brightness but influenced by shifts in hue.

The *∆*E values did not show a statistically significant difference between the enamel and dentin groups, indicating that the color match of OMNICHROMA composite is consistent across different cavity depths. The lack of a significant linear relationship between initial tooth color parameters and *∆*E suggests that predicting color match based on initial tooth color alone is not feasible.

In this study, *∆*E measurements in all samples ranged from 0.7 to 3.7. In Saegusa et al.'s [14] study, the *∆*E range measured was 1.9–3.3. In both studies, the upper limit of the spectrum was close to the clinically acceptable threshold. However, in this study, the upper limit of *∆*E was higher at 3.7. This slight difference could be attributed to the fact that the samples in the Saegusa study were acrylic teeth. Naturally, the *∆*E threshold perceptible by the general population takes precedence over the clinician's preference, which placed the composite in the acceptable range for nonprofessional observers. Although the average *∆*E obtained was close to the threshold perceived by clinicians, the clinically perceptible *∆*E threshold is considered to be 2.2 for clinicians [16] and 3.3 for nonprofessional observers [17].

Tooth color is primarily determined by dentin, not enamel, which has only a minor influence on tooth color but plays a significant role in brightness [7]. In this study, two cavities with different depths were prepared: one at the enamel level and the other at the dentin level. The results confirmed that in deep dentin cavities, the highest degree of mismatch was due to differences in yellow–blue color (*p*  < 0.001) and brightness (*p*=0.989) (Table 1). Since dentin tissue is the main factor determining color, enamel has a lesser impact. However, in enamel cavities, color matching was weaker in terms of brightness, related to enamel's optical properties. The analysis showed that in shallow enamel cavities, the composite became darker compared to the tooth color and shifted toward green and blue, though the shift toward blue was not statistically significant (*p*=0.775). This result contrasts with a study by Morsy, Gamal, and Riad [9], where 1.5 mm depth cavities in the cervical area showed that the composite color became brighter and shifted toward green and blue. This difference may be due to the cavity location, where enamel thickness is less in the cervical area.

In this study, predicting the degree of color match of the OMNICHROMA composite before restoration was challenging. As shown in Table 4, no linear relationship was found between the initial tooth color and optical factors with the degree of color match. This contrasts with a study by Iyer et al. [18], which claimed that increasing L values resulted in a decrease in color matching. This discrepancy may be due to different devices used to measure color, a lower number of samples with darker colors, and improvements in the composite formulation by the manufacturer.

Based on the results, although OMNICHROMA composite color match with tooth structure is within the clinically acceptable range, the ideal *∆*E for tooth color matching is 1 [17]. This suggests that when esthetic and color matching of composite resin to tooth structure is crucial, conventional composites are preferred for restoration [18]. Furthermore, with the introduction of body and enamel composites, especially in esthetic areas, a single-color composite may not effectively mimic the natural multilayered structure of teeth and yield excellent esthetic results. However, OMNICHROMA is a suitable replacement when different colors of conventional composites are not available.

As a limitation of this study, it is better to investigate the color matching of OMNICHROMA in the oral cavity. Dental materials may behave differently over time when placed in the oral cavity due to factors such as temperature, pH, salivary enzymes, and the presence of bacteria. Additionally, it is necessary to investigate the effect of the blocker composite provided with OMNICHROMA on color matching.

## 9. Conclusion

The color match (*∆*E) of OMNICHROMA composite, as an intelligent chromatic composite system, fell within the clinically acceptable range for enamel and dentin cavities.

## Figures and Tables

**Table 1:** Mean optical parameters of different groups.

Depth	L	a	b
Enamel			
Tooth surface			
Mean	72.47	1.46	20.71
Standard deviation (SD)	3.05	1.06	3.23
Restoration surface			
Mean	71.26	0.46	20.64
SD	2.78	0.98	2.53
*Δ*	−1.20	−0.99	−0.70
*p*-Value	<0.001	<0.001	0.775
Dentin			
Tooth surface			
Mean	72.070	1.46	20.06
SD	3.46	1.11	4.18
Restoration surface			
Mean	72.073	0.61	20.57
SD	2.95	0.97	3.08
*Δ*	0.003	−0.85	0.50
*p*-Value	0.989	<0.001	0.137

**Table 2:** Comparison of *∆*E based on depth of preparation.

Depth	Mean	SD	*p*-Value
*∆*E			
Enamel	2.39	0.61	0.719
Dentin	2.33	0.80	

**Table 3:** Comparison of the absolute difference in optical parameters at two depths.

Depth	Mean	SD	*p*-Value
*∆*L			
Enamel	1.60	0.81	0.70
Dentin	1.04	0.81	
*∆*a			
Enamel	0.99	0.43	0.29
Dentin	0.85	0.37	
*∆*b			
Enamel	1.13	0.67	0.12
Dentin	1.60	0.95	

**Table 4:** Examination of the correlation coefficient between *∆*E and the absolute difference in each optical parameter, differentiated by depth.

Cavity depth	*p*-Value	Correlation
Enamel		
*∆*L	<0.001	0.78
*∆*a	0.55	0.11
*∆*b	0.09	0.31
Dentin		
*∆*L	0.002	0.53
*∆*a	0.04	0.36
*∆*b	<0.001	0.79

**Table 5:** Examination of the correlation coefficient between *∆*E and the initial value of each optical parameter, differentiated by depth.

Depth	*p*-Value	Correlation
Enamel		
L_1_	0.41	0.16
a_1_	0.95	−0.012
b_1_	0.57	−0.11
Dentin		
L_2_	0.40	0.16
a_2_	0.29	−0.20
b_2_	0.38	−0.17

**Table 6:** Frequency distribution of *∆*E based on less than, equal to, or greater than 3.3 in enamel and dentin groups.

*∆*E value	Depth		Total
	Dentin	Enamel	
*∆*E			
*∆*E < 3.3			
Number	24	27	51
Frequency (%)	80.0	90.0	85.0
*∆*E ≥ 3.3			
Number	6	3	9
Frequency (%)	20.0	10.0	15.0
Total			
Number	30	30	60
Frequency (%)	100.0	100.0	100.0

## Data Availability

The data supporting the results of this study are available upon reasonable request from the corresponding author.
